# Coupled fixed point theorems in *G*_*b*_-metric space satisfying some rational contractive conditions

**DOI:** 10.1186/s40064-016-2925-7

**Published:** 2016-08-05

**Authors:** Bulbul Khomdram, Yumnam Rohen, Thokchom Chhatrajit Singh

**Affiliations:** Department of Mathematics, NIT Manipur, 795004 Imphal, India

**Keywords:** Common fixed points, *G*_*b*_-metric space, Rational contractive, 47H10, 54H25, 47H09

## Abstract

In this paper we prove the existence and uniqueness of couple fixed point theorems for three mappings satisfying some new rational contractive conditions. We prove our results in the frame work of *G*_*b*_-metric space which is recently introduced by Aghajani et al. (Filomat 28(6):1087–1101, 2014). Illustrative example is also given to support our result.

## Introduction and preliminaries

A metric space is a set X together with a function *d* (called a metric or “distance function”) which assigns a real number *d*(*x*, *y*) to every pair x, y belonging to X satisfying the properties (or axioms):

1. $$d(x, y) \ge 0$$ and $$d(x, y)=0$$ iff $$x = y$$
2. $$d(x, y)=d(y, x)$$,
3. $$d(x, y) + d(y, z)\ge d(x, z)$$.

The pair (*X*, *d*) is called a metric space.

Metric fixed point theory is one of the most important and fundamental area of analysis. Due to this a flood of research work have been generated from this area. As a part of this study generalisation of metric space becomes one of the most interesting topic in which many researchers have devoted and continued working. Since the introduction of metric space by Frachet, there is a lot of generalisation of this space. Some of them which can be mentioned are 2-metric space, *D*-metric space, *G*-metric space, cone metric space, fuzzy metric space, Menger space, probabilitic metric space, partial metric space, quasi metric space, *b*-metric space, multiplicative metric space, modular metric space, cyclic metric space, *S*-metric space, *b*-cone metric space etc.

In a recent paper, Aghajani et al. (2014) introduced a new generalisation of metric space. They used the concepts of both *G*-metric and *b*-metric and generated the new definition and named it as *G*_*b*_-metric space. They also pointed out that the class of *G*_*b*_-metric space is effectively larger than that of *G*-metric space and *G*-metric space becomes a particular case of *G*_*b*_-metric space. They claimed that every *G*_*b*_-metric space is topologically equivalent to a *b*-metric space. For more results on *b*-metric space one can study the research papers of Malhotra and Bansal (2015), Czerwik (1993, 1998), Hussain et al. (2013), Singh and Singh (2015) and references there in. Results of *G*_*b*_-metric also can be found in the research papers of Mustafa et al. (2011, 2013a, b), Sedghi et al. (2014), Shahkoohi et al. (2011), Roshan et al. (2014) and references there in.

The study of fixed points for more than one dimension is becoming an interest for many researchers for the last many years. This concept was first initiated by Guo and Lakshmikantham (1987) by introducing the definition of coupled fixed point in the year 1987. After a gap of about twenty years Bhaskar and Lakshmikantham (2006) in the year 2006 proved a fixed point theorem for a mixed monotone mapping in a metric space endowed with partial order. Since then large number of research papers came out about coupled fixed point. This concept is further extended to tripled fixed point by Berinde and Borcut (2011) and to quadrupled fixed point by Karapinar. For more results on multidimensional fixed point one can see the research papers in Kutbi et al. (2013), Mustafa et al. (2011, 2013a, b), Sedghi et al. (2014), Shahkoohi et al. (2011), Guo and Lakshmikantham (1987), Berinde and Borcut (2011), Abbas et al. (2010), Karapinar (2011), Long et al. (2012), Kadelburg and Radenovic (2012), Batra and Vashistha (2013), Batra et al. (2014), Karapinar and Turkoglu (2010), Shantanawi (2010), Aghajani et al. (2012), Mehta and Joshi (2010), Malhotra and Bansal (2015), Karapinar, Bhaskar and Lakshmikantham (2006), Singh and Singh (2014) and reference therein.

In our present study we prove some unique coupled common fixed point theorems for three mappings satisfying some new rational contractive conditions in $G_{b}$-metric space. Our result is a new result of this type in the setting of $G_{b}$-metric space.

Following definition was given by Mustafa et al. (2011, 2013b)

### **Definition 1**

(Mustafa et al. 2013b) Let X be a nonempty set and $G:X^{3}\rightarrow \mathbb {R^{+}}$ be a function satisfying the following properties:

1. $G(x,y,z)=0$ if and only if $x=y=z$;
2. $0<G(x, x, y)$ for all $x, y\in X$ with $x \ne y$;
3. $G(x, x, y)\le G(x, y, z)$ for all $x,y,z\in X$ with $z \ne y$;
4. $G(x, y, z)=G(x, z, y)=G(y, z, x)=\dots$ (symmetry in all three variables);
5. $G(x, y, z)\le G(x, a, a) + G(a, y, z)$ for all $x,y,z,a\in X$ (rectangle inequality).

Then the function *G* is called a $G-$metric on *X* and the pair (*X*, *G*) is called a G-metric space.

Following definition was given by Bakhtin (1989), Czerwik (1993, 1998)

### **Definition 2**

(Malhotra and Bansal 2015) Let X be a (nonempty) set and $s\ge 1$ a given real number. A function $d: X\times X\rightarrow \mathbb {R^{+}}$ (nonnegative real numbers) is called a $b-$metric provided that, for all $x,y,z \in X$, following conditions are satisfied:

1. $d(x, y) = 0$ if and only if $x = y$;
2. $$d(x, y) = d(y, x);$$
3. $$d(x, z) \le s[d(x, y) + d(y, z)]$$

The pair (*X*, *d*) is called a b-metric space with parameter s.

Following definition was given by Aghajani et al. (2014)

### **Definition 3**

(Aghajani et al. 2014) Let *X* be a nonempty set and $s\ge 1$ be a given real number. Suppose that a mapping $G:X\times X\times X\rightarrow R^{+}$ satisfies:

$$(G_{b}1)$$
$$G(x,y,z) =0$$ if $$x=y=z$$,
$$(G_{b}2)$$
$0<G(x,x,y)$ for all $x,y\in X$ with $x\not =y$,
$$(G_{b}3)$$
$G(x,x,y)\le G(x,y,z)$ for all $x,y,z\in X$ with $y\not =z$,
$$(G_{b}4)$$
$G(x,y,z)=G(p[x,y,z])$, where *p* is a permutation of *x*, *y*, *z* (symmetry),
$$(G_{b}5)$$
$G(x,y,z)=s[G(x,a,a)+G(a,y,z)]$ for all $x,y,z,a\in X$ (rectangle inequality).

Then *G* is called a generalized *b*-metric and pair (*X*, *G*) is called a generalized *b*-metric space or $G_{b}$-metric space.

Aghajani et al. (2014) remarked that the class of $G_{b}$-metric space is effectively larger than that of *G*-metric spaces given in Mustafa et al. (2013a). Following example given by Aghajani et al. (2014) shows that a $G_{b}$-metric on *X* need not be a *G*-metric on *X*.

### *Example 4*

(Aghajani et al. 2014) Let (*X*, *G*) be a *G*-metric space, and $G_{*}(x,y,z)=G(x,y,z)^{p}$, where $p>1$ is a real number. Note that $G_{*}$ is a $G_{b}$-metric with $s=2^{p-1}$.

Also in the above example, $(X, G_{*})$ is not necessarily a *G*-metric space. For example, let $X ={\mathbb {R}}$ and *G*-metric *G* be defined by

$$\begin{aligned} G(x,y,z)=\frac{1}{3}(|x-y|+|y-z|+|x-z|), \end{aligned}$$

for all $x,y,z\in {\mathbb {R}}$. Then $G_{*}(x,y,z)^{2}=\frac{1}{9}(|x-y|+|y-z|+|x-z|)^{2}$ is a $G_{b}$-metric on ${\mathbb {R}}$ with $s=2^{2-1}=2$, but it is not a *G*-metric on ${\mathbb {R}}$. To see this, let $x=3$, $y=5$, $z=7$, $a=\frac{7}{2}$ we get, $G_{*}(3,5,7)=\frac{64}{9}$, $G_{*}(3,\frac{7}{2},\frac{7}{2})=\frac{1}{9}$, $G_{*}\left( \frac{7}{2},5,7\right) =\frac{49}{9}$, so $G_{*}(3,5,7)=\frac{64}{9} \nleq \frac{50}{9}=G_{*}(3,\frac{7}{2},\frac{7}{2})+G_{*}\left( \frac{7}{2},5,7\right)$.

Following definitions and propositions in $G_{b}$-metric space are given in Aghajani et al. (2014).

### **Definition 5**

(Aghajani et al. 2014) A $G_{b}$-metric *G* is said to be symmetric if $G(x,y,y)=G(y,x,x)$ for all $x,y\in X$.

### **Definition 6**

(Aghajani et al. 2014) Let (*X*, *G*) be a $G_{b}$-metric space then for $x_{0}\in X$, $r>0$, the $G_{b}$-ball with center $x_{0}$ and radius *r* is

$$\begin{aligned} B_{G}(x_{0},r)=\{y\in X|G(x_{0},y,y)< r\} \end{aligned}$$

For example, let $X={\mathbb {R}}$ and consider the $G_{b}$-metric *G* defined by

$$\begin{aligned} G(x,y,z)=\frac{1}{9}(|x-y|+|y-z|+|x-z|)^{2} \end{aligned}$$

for all $x,y,z \in {\mathbb {R}}$. Then

$$\begin{aligned} B_{G}(3,4)& = \{y\in X:\, G(3,y,y)<4\} \\& = \left\{y\in X:\frac{1}{9}(|y-3|+|y-3|)^{2}<4\right\} \\& = \{y\in X:\,\,|y-3|^{2}<9\} \\& = (0,6). \end{aligned}$$

### **Proposition 7**

(Aghajani et al. 2014) *LetXbe a*$G_{b}$-*metric space, then for each*$x,y,z,a \in X$*it follows that:*

1. if $$G(x,y,z)=0$$ then $$x=y=z$$,
2. $$G(x,y,z)\le s(G(x,x,y)+G(x,x,z))$$,
3. $$G(x,y,y)\le 2sG(y,x,x)$$,
4. $$G(x,y,z)\le s(G(x,a,z)+G(a,y,z))$$

### **Definition 8**

(Aghajani et al. 2014) Let *X* be a $G_{b}$-metric space, we define $d_{G}(x, y)=G(x,y,y)+G(x,x,y)$, it is easy to see that $d_{G}$ defines a *b*-metric on *X*, which we call it *b*-metric associated with *G*.

### **Proposition 9**

(Aghajani et al. 2014) *LetXbe a*$G_{b}$-*metric space, then for any*$x_{0}\in X$*and*$r>0$, *if*$y\in B_{G}(x_{0},r)$*then there exists a*$\delta >0$*such that*$B_{G}(y,\delta )\subseteq B_{G}(x_{0}, r)$.

### **Proposition 10**

(Aghajani et al. 2014) *LetXbe a*$G_{b}$-*metric space, then for any*$x_{0}\in X$*and*$r>0$, *we have*

$$\begin{aligned} B_{G}\left( x_{0},\frac{r}{2s+1}\right) \subseteq B_{d_{G}}(x_{0}, r) \subseteq B_{G}(x_{0}, r) \end{aligned}$$

*Thus every*$G_{b}$*-metric space is topologically equivalent to ab-metric space. This allows us to readily transport many concepts and results fromb-metric spaces into*$G_{b}$*-metric space setting.*

### **Definition 11**

(Aghajani et al. 2014) Let *X* be a $G_{b}$-metric space. A sequence $\{x_{n}\}$ in *X* is said to be:

1. $G_{b}$-Cauchy sequence if, for each $\varepsilon >0$, there exists a positive integer $n_{0}$ such that, for all $m,n,l\ge n_{0}, G(x_{n}, x_{m}, x_{l})<\varepsilon$;
2. $G_{b}$-convergent to a point $x\in X$ if, for each $\varepsilon > 0$, there exists a positive integer $n_{0}$ such that, for all $m,n=n_{0}, G(x_{n},x_{m},x)<\varepsilon$.

### **Proposition 12**

(Aghajani et al. 2014) *LetXbe a*$G_{b}$-*metric space, then following statements are equivalent:*

1. *the sequence*$\{x_{n}\}$*is*$G_{b}$-*Cauchy*.
2. *for any*$\varepsilon >0$, *there exists*$n_{0}\in N$*such that*$G(x_{n},x_{m},x_{m})<\varepsilon$, *for all*$m,n\ge n_{0}$.

### **Proposition 13**

(Aghajani et al. 2014) *LetXbe a*$G_{b}$-*metric space, then following statements are equivalent*:

1. $\{x_{n}\}$ is $G_{b}$-*convergent* to *x*.
2. $$G(x_{n},x_{n},x)\rightarrow 0$$ as $$n\rightarrow +\infty$$.
3. $$G(x_{n},x,x)\rightarrow 0$$ as $$n\rightarrow +\infty$$.

### **Definition 14**

(Aghajani et al. 2014) A $G_{b}$-metric space *X* is called $G_{b}$-complete if every $G_{b}$-Cauchy sequence is $G_{b}$-convergent in *X*.

### **Definition 15**

(Kutbi et al. 2013) Let *X* be a nonempty set. Then $(X,G,\preceq )$ is called partially ordered $G_{b}$-metric space if *G* is a $G_{b}$-metric on a partially ordered set $(X,\preceq )$.

## Main results

Now we prove the following theorem.

### **Theorem 16**

*Let* (*X*, *G*) *be a complete symmetric*$G_{b}$-*metric space with parameter*$s\ge 1$*and let the mappings*$S,T,R:X^{2}\rightarrow X$*satisfying*

1 $$\begin{aligned} G(S(x,y),T(u,v),R(a,b))& \le \alpha _{1}\frac{G(x,u,a)+G(y,v,b)}{2} \nonumber \\& \quad +\alpha _{2}\frac{G(S(x,y),T(u,v),R(a,b))G(x,u,a)}{1+G(x,u,a)+G(y,v,b)} \nonumber \\& \quad +\alpha _{3}\frac{G(S(x,y),T(u,v),R(a,b))G(y,v,b)}{1+G(x,u,a)+G(y,v,b)} \nonumber \\& \quad +\alpha _{4}\frac{G(x,x,S(x,y))G(x,u,a)}{1+G(x,u,a)+G(y,v,b)} \nonumber \\& \quad +\alpha _{5}\frac{G(x,x,S(x,y))G(y,v,b)}{1+G(x,u,a)+G(y,v,b)} \nonumber \\& \quad +\alpha _{6}\frac{G(u,u,T(u,v))G(x,u,a)}{1+G(x,u,a)+G(y,v,b)} \nonumber \\& \quad +\alpha _{7}\frac{G(u,u,T(u,v))G(y,v,b)}{1+G(x,u,a)+G(y,v,b)} \nonumber \\& \quad +\alpha _{8}\frac{G(a,a,R(a,b))G(x,u,a)}{1+G(x,u,a)+G(y,v,b)} \nonumber \\& \quad +\alpha _{9}\frac{G(a,a,R(a,b))G(y,v,b)}{1+G(x,u,a)+G(y,v,b)} \end{aligned}$$

*for all*$x,y,u,v,a,b \in X$*and*$\alpha _{1},\alpha _{2},...,\alpha _{9}\ge 0$*with*$\alpha _{1}+\alpha _{2}+\alpha _{3}+2(\alpha _{4}+\alpha _{5})+\alpha _{6}+\alpha _{7}+\alpha _{8}+\alpha _{9}<1$. *ThenS*, *TandRhave a unique common coupled fixed point inX*.

### *Proof*

Let $x_{0},y_{0}\in X$ be arbitrary points.

Define

$$\begin{aligned} x_{3k+1}& = S(x_{3k},y_{3k}),y_{3k+1} = S(y_{3k},x_{3k}) \\ x_{3k+2}& = T(x_{3k+1},y_{3k+1}),y_{3k+2} = T(y_{3k+1},x_{3k+1}) \\ x_{3k+3}& = R(x_{3k+2},y_{3k+2}),y_{3k+3} = R(y_{3k+2},x_{3k+2}) \end{aligned}$$

for $$k=0,1,2,3,...$$. Then

$$\begin{aligned} G(x_{3k+1},x_{3k+2},x_{3k+3})& = G(S(x_{3k},y_{3k}),T(x_{3k+1},y_{3k+1}),R(x_{3k+2},y_{3k+2}))\\& \le \alpha _{1}\frac{G(x_{3k},x_{3k+1},x_{3k+2})+G(y_{3k},y_{3k+1},y_{3k+2})}{2} \\& \quad +\alpha _{2}\frac{G(S(x_{3k},y_{3k}),T(x_{3k+1},y_{3k+1}),R(x_{3k+2},y_{3k+2}))G(x_{3k},x_{3k+1},x_{3k+2})}{1+G(x_{3k},x_{3k+1},x_{3k+2})+G(y_{3k},y_{3k+1},y_{3k+2})} \\& \quad +\alpha _{3}\frac{G(S(x_{3k},y_{3k}),T(x_{3k+1},y_{3k+1}),R(x_{3k+2},y_{3k+2}))G(y_{3k},y_{3k+1},y_{3k+2})}{1+G(x_{3k},x_{3k+1},x_{3k+2})+G(y_{3k},y_{3k+1},y_{3k+2})} \\& \quad +\alpha _{4}\frac{G(x_{3k},x_{3k},S(x_{3k},y_{3k}))G(x_{3k},x_{3k+1},x_{3k+2})}{1+G(x_{3k},x_{3k+1},x_{3k+2})+G(y_{3k},y_{3k+1},y_{3k+2})} \\& \quad +\alpha _{5}\frac{G(x_{3k},x_{3k},S(x_{3k},y_{3k}))G(y_{3k},y_{3k+1},y_{3k+2})}{1+G(x_{3k},x_{3k+1},x_{3k+2})+G(y_{3k},y_{3k+1},y_{3k+2})} \\& \quad +\alpha _{6}\frac{G(x_{3k+1},x_{3k+1},T(x_{3k+1},y_{3k+1}))G(x_{3k},x_{3k+1},x_{3k+2})}{1+G(x_{3k},x_{3k+1},x_{3k+2})+G(y_{3k},y_{3k+1},y_{3k+2})} \\& \quad +\alpha _{7}\frac{G(x_{3k+1},x_{3k+1},T(x_{3k+1},y_{3k+1}))G(y_{3k},y_{3k+1},y_{3k+2})}{1+G(x_{3k},x_{3k+1},x_{3k+2})+G(y_{3k},y_{3k+1},y_{3k+2})} \\& \quad +\alpha _{8}\frac{G(x_{3k+2},x_{3k+2},R(x_{3k+2},y_{3k+2}))G(x_{3k},x_{3k+1},x_{3k+2})}{1+G(x_{3k},x_{3k+1},x_{3k+2})+G(y_{3k},y_{3k+1},y_{3k+2})} \\& \quad +\alpha _{9}\frac{G(x_{3k+2},x_{3k+2},R(x_{3k+2},y_{3k+2}))G(y_{3k},y_{3k+1},y_{3k+2})}{1+G(x_{3k},x_{3k+1},x_{3k+2})+G(y_{3k},y_{3k+1},y_{3k+2})} \\& = \alpha _{1}\frac{G(x_{3k},x_{3k+1},x_{3k+2})+G(y_{3k},y_{3k+1},y_{3k+2})}{2} \\& \quad +\alpha _{2}\frac{G(x_{3k+1},x_{3k+2},x_{3k+3})G(x_{3k},x_{3k+1},x_{3k+2})}{1+G(x_{3k},x_{3k+1},x_{3k+2})+G(y_{3k},y_{3k+1},y_{3k+2})} \\& \quad +\alpha _{3}\frac{G(x_{3k+1},x_{3k+2},x_{3k+3})G(y_{3k},y_{3k+1},y_{3k+2})}{1+G(x_{3k},x_{3k+1},x_{3k+2})+G(y_{3k},y_{3k+1},y_{3k+2})} \\& \quad +\alpha _{4}\frac{G(x_{3k},x_{3k},x_{3k+1})G(x_{3k},x_{3k+1},x_{3k+2})}{1+G(x_{3k},x_{3k+1},x_{3k+2})+G(y_{3k},y_{3k+1},y_{3k+2})} \\& \quad +\alpha _{5}\frac{G(x_{3k},x_{3k},x_{3k+1})G(y_{3k},y_{3k+1},y_{3k+2})}{1+G(x_{3k},x_{3k+1},x_{3k+2})+G(y_{3k},y_{3k+1},y_{3k+2})} \\& \quad +\alpha _{6}\frac{G(x_{3k+1},x_{3k+1},x_{3k+2})G(x_{3k},x_{3k+1},x_{3k+2})}{1+G(x_{3k},x_{3k+1},x_{3k+2})+G(y_{3k},y_{3k+1},y_{3k+2})} \\& \quad +\alpha _{7}\frac{G(x_{3k+1},x_{3k+1},x_{3k+2})G(y_{3k},y_{3k+1},y_{3k+2})}{1+G(x_{3k},x_{3k+1},x_{3k+2})+G(y_{3k},y_{3k+1},y_{3k+2})} \\& \quad +\alpha _{8}\frac{G(x_{3k+2},x_{3k+2},x_{3k+3})G(x_{3k},x_{3k+1},x_{3k+2})}{1+G(x_{3k},x_{3k+1},x_{3k+2})+G(y_{3k},y_{3k+1},y_{3k+2})} \\& \quad +\alpha _{9}\frac{G(x_{3k+2},x_{3k+2},x_{3k+3})G(y_{3k},y_{3k+1},y_{3k+2})}{1+G(x_{3k},x_{3k+1},x_{3k+2})+G(y_{3k},y_{3k+1},y_{3k+2})} \end{aligned}$$

which implies that

$$\begin{aligned}&(1-\alpha _{2}-\alpha _{3})G(x_{3k+1},x_{3k+2},x_{3k+3}) \le \frac{\alpha _{1}}{2}G(x_{3k},x_{3k+1},x_{3k+2}) \\ & \quad +\frac{\alpha _{1}}{2}G(y_{3k},y_{3k+1},x_{3k+2}) \\&\quad +(\alpha _{4}+\alpha _{5})G(x_{3k},x_{3k+1},x_{3k+2})+(\alpha _{6}+\alpha _{7})G(x_{3k+1},x_{3k+2},x_{3k+3})\\&\quad +(\alpha _{8}+\alpha _{9})G(x_{3k+1},x_{3k+2},x_{3k+3})\\&\Rightarrow (1-\alpha _{2}-\alpha _{3}-\alpha _{6}-\alpha _{7} -\alpha _{8}-\alpha _{9})G(x_{3k+1},x_{3k+2},x_{3k+3})\\ & \le \left( \frac{\alpha _{1}}{2}+\alpha _{4}+\alpha _{5}\right) G(x_{3k},x_{3k+1},x_{3k+2})\\&\quad +\frac{\alpha _{1}}{2}G(y_{3k},y_{3k+1},y_{3k+2}) \end{aligned}$$

Therefore,

2 $$\begin{aligned} G(x_{3k+1},x_{3k+2},x_{3k+3})& \le \frac{\left( \frac{\alpha _{1}}{2}+\alpha _{4}+\alpha _{5}\right) G(x_{3k},x_{3k+1},x_{3k+2})}{1-\alpha _{2}-\alpha _{3}-\alpha _{6}-\alpha _{7}-\alpha _{8}-\alpha _{9}} \nonumber \\& \quad + \frac{\frac{\alpha _{1}}{2}G(y_{3k},y_{3k+1},y_{3k+2})}{1-\alpha _{2}-\alpha _{3}-\alpha _{6}-\alpha _{7}-\alpha _{8}-\alpha _{9}} \end{aligned}$$

Proceeding similarly one can prove that

3 $$\begin{aligned} G(y_{3k+1},y_{3k+2},y_{3k+3})& \le \frac{\left( \frac{\alpha _{1}}{2}+\alpha _{4}+\alpha _{5}\right) G(y_{3k},y_{3k+1},y_{3k+2})}{1-\alpha _{2}-\alpha _{3}-\alpha _{6}-\alpha _{7}-\alpha _{8}-\alpha _{9}} \nonumber \\& \quad + \frac{\frac{\alpha _{1}}{2}G(x_{3k},x_{3k+1},x_{3k+2})}{1-\alpha _{2}-\alpha _{3}-\alpha _{6}-\alpha _{7}-\alpha _{8}-\alpha _{9}} \end{aligned}$$

Adding (2) and (3) we have

$$\begin{aligned}&G(x_{3k+1},x_{3k+2},x_{3k+3})+G(y_{3k+1},y_{3k+2},y_{3k+3}))\\&\quad \le \frac{\alpha _{1}+2(\alpha _{4}+\alpha _{5})}{1-\alpha _{2}-\alpha _{3}-\alpha _{6}-\alpha _{7}-\alpha _{8}-\alpha _{9}}[G(x_{3k},x_{3k+1},x_{3k+2}) +G(y_{3k},y_{3k+1},y_{3k+2})] \end{aligned}$$

Therefore

$$\begin{aligned}&G(x_{3k+1},x_{3k+2},x_{3k+3})+G(y_{3k+1},y_{3k+2},y_{3k+3}))\\&\quad \le h[G(x_{3k},x_{3k+1},x_{3k+2})+G(y_{3k},y_{3k+1},y_{3k+2})] \end{aligned}$$

where

$$\begin{aligned} h=\frac{\alpha _{1}+2(\alpha _{4}+\alpha _{5})}{1-\alpha _{2}-\alpha _{3}-\alpha _{6}-\alpha _{7}-\alpha _{8}-\alpha _{9}}<1 \end{aligned}$$

Also, we can show that

$$\begin{aligned}&G(x_{3k+2},x_{3k+3},x_{3k+4})+G(y_{3k+2},y_{3k+3},y_{3k+4}))\\&\quad \le h[G(x_{3k+1},x_{3k+2},x_{3k+3})+G(y_{3k+1},y_{3k+2},y_{3k+3})] \\&\quad \le h^{2} [G(x_{3k},x_{3k+1},x_{3k+2})+G(y_{3k},y_{3k+1},y_{3k+2})] \end{aligned}$$

Continuing this way, we have

$$\begin{aligned}&G(x_{n},x_{n+1},x_{n+2})+G(y_{n},y_{n+1},y_{n+2})\\&\quad \le h[G(x_{n-1},x_{n},x_{n+1})+G(y_{n-1},y_{n},y_{n+1})]\\&\quad \le h^{2}[G(x_{n-2},x_{n-1},x_{n})+G(y_{n-2},y_{n-1},y_{n})]\\&\quad \le \cdots \le h^{n}[G(x_{0},x_{1},x_{2})+G(y_{0},y_{1},y_{2})] \end{aligned}$$

If $$G(x_{n},x_{n+1},x_{n+2})+G(y_{n},y_{n+1},y_{n+2})=G_{n}$$, then $$G_{n}\le hG_{n-1}\le h^{2}G_{n-2}\le \cdots \le h^{n}G_{0}$$.

By property (3) of Definition 1, we have

$$\begin{aligned} G(x_{n},x_{n+1},x_{n+1})+G(y_{n},y_{n+1},y_{n+1}) \le G_{n} \le h^{n}G_{0} \end{aligned}$$

For $$m>n$$,

$$\begin{aligned}&G(x_{n},x_{m},x_{m})+G(y_{n},y_{m},y_{m})\\&\quad \le s[G(x_{n},x_{n+1},x_{n+1})+G(x_{n+1},x_{m},x_{m})+G(y_{n},y_{n+1},y_{n+1})+G(y_{n+1},y_{m},y_{m})]\\&\quad \le s[G(x_{n},x_{n+1},x_{n+1})+G(y_{n},y_{n+1},y_{n+1})]+s^{2}[G(x_{n+1},x_{n+2},x_{n+2})+G(y_{n+1},y_{n+2},y_{n+2})]+ \cdots \\&\qquad +s^{m-n}[G(x_{m-1},x_{m},x_{m})+G(y_{m-1},y_{m},y_{m})]\\&\quad \le sh^{n}G_{0}+s^{2}h^{n+1}G_{0}+ \cdots +s^{m-n}h^{m-1}G_{0}\\&\quad <sh^{n}[1+sh+(sh)^{2}+ \cdots ]G_{0}\\&\quad =\frac{sh^{n}}{1-sh} G_{0}\rightarrow 0 \quad {\text{as }} n\rightarrow \infty \end{aligned}$$

which shows that $\{x_{n}\}$ and $\{y_{n}\}$ are Cauchy sequences in *X*. As *X* is complete $G_{b}$-metric space, so there exists $x,y\in X$ such that $x_{n}\rightarrow x$ and $y_{n}\rightarrow y$ as $n\rightarrow \infty$.

Now we will prove that $x=S(x,y)$ and $y=S(y,x)$. On contrary suppose that $x\ne S(x,y)$ and $y\ne S(y,x)$. Then $G(x,S(x,y),S(x,y))=l_{1}>0$ and $G(y,y,S(y,x))=l_{2}>0$.

Using inequality (1) we have

$$\begin{aligned} l_{1}& = G(x,S(x,y),S(x,y)) \\& \le s[G(x,x_{n+1},x_{n+1})+G(x_{n+1},S(x,y),S(x,y))] \\& \le sG(x,x_{n+1},x_{n+1})+s\left[ \alpha _{1}\frac{G(x_{n},x,x)+G(y_{n},y,y)}{2}\right. \\& \quad \left. +\;\alpha _{2}\frac{G(S(x_{n},y_{n}),S(x,y),S(x,y))G(x_{n},x,x)}{1+G(x_{n},x,x)+G(y_{n},y,y)} +\alpha _{3}\frac{G(S(x_{n},y_{n}),S(x,y),S(x,y))G(y_{n},y,y)}{1+G(x_{n},x,x)+G(y_{n},y,y)} \right. \\& \quad \left. +\;\alpha _{4}\frac{G(x_{n},x_{n},S(x_{n},y_{n}))G(x_{n},x,x)}{1+G(x_{n},x,x)+G(y_{n},y,y)} +\alpha _{5}\frac{G(x_{n},x_{n},S(x_{n},y_{n}))G(y_{n},y,y)}{1+G(x_{n},x,x)+G(y_{n},y,y)}\right. \\& \quad \left. +\;\alpha _{6}\frac{G(x,x,S(x,y))G(x_{n},x,x)}{1+G(x_{n},x,x)+G(y_{n},y,y)} +\alpha _{7}\frac{G(x,x,S(x,y))G(y_{n},y,y)}{1+G(x_{n},x,x)+G(y_{n},y,y)}\right. \\& \quad \left. +\;\alpha _{8}\frac{G(x,x,S(x,y))G(x_{n},x,x)}{1+G(x_{n},x,x)+G(y_{n},y,y)} +\alpha _{9}\frac{G(x,x,S(x,y))G(y_{n},y,y)}{1+G(x_{n},x,x)+G(y_{n},y,y)} \right] . \end{aligned}$$

Since $\{x_{n}\}$ and $\{y_{n}\}$ are convergent to x and y, therefore by taking limits as $n\rightarrow \infty$ we get $l_{1}\le 0$, which is a contradiction, so $G(x,S(x,y),S(x,y))=0$ which gives $x=S(x,y)$.

Similarly, we can prove that $y=S(y,x)$. Also, we can prove that $x=T(x,y)$ and $y=T(y,x)$. Similarly $x=R(x,y)$ and $y=R(y,x)$. Then (*x*, *y*) is a Common coupled fixed point of *S*, *T* and *R*.

In order to prove the uniqueness of the coupled fixed point, if possible let (*p*, *q*) be the second common coupled fixed point of *S*, *T* and *R*.

Then by using inequality (1), we have

4 $$\begin{aligned} G(x,p,p)& = G(S(x,y),T(p,q),R(p,q)) \nonumber \\& \le \alpha _{1}\frac{G(x,p,p)+G(y,q,q)}{2}+\alpha _{2}\frac{G(S(x,y),T(p,q),R(p,q))G(x,p,p)}{1+G(x,p,p)+G(y,q,q)}\nonumber \\& \quad +\alpha _{3}\frac{G(S(x,y),T(p,q),R(p,q))G(y,q,q)}{1+G(x,p,p)+G(y,q,q)}\nonumber \\& \quad +\alpha _{4}\frac{G(x,x,S(x,y))G(x,p,p)}{1+G(x,p,p)+G(y,q,q)}+\alpha _{5}\frac{G((x,x,S(x,y)))G(y,q,q)}{1+G(x,p,p)+G(y,q,q)}\nonumber \\& \quad +\alpha _{6}\frac{G(p,p,T(p,q))G(x,p,p)}{1+G(x,p,p)+G(y,q,q)}+\alpha _{7}\frac{G(p,p,T(p,q))G(y,q,q)}{1+G(x,p,p)+G(y,q,q)}\nonumber \\& \quad +\alpha _{8}\frac{G(p,p,R(p,q))G(x,p,p)}{1+G(x,p,p)+G(y,q,q)}+\alpha _{9}\frac{G(p,p,R(p,q))G(y,q,q)}{1+G(x,p,p)+G(y,q,q)}\nonumber \\& = \frac{\alpha _{1}}{2}[G(x,p,p)+G(y,q,q)]\nonumber \\& \quad +\alpha _{2}\frac{G(x,p,p)G(x,p,p)}{1+G(x,p,p)+G(y,q,q)}+\alpha _{3}\frac{G(x,p,p)G(y,p,p)}{1+G(x,p,p)+G(y,q,q)}\nonumber \\& \quad +\alpha _{4}\frac{G(x,x,x)G(x,p,p)}{1+G(x,p,p)+G(y,q,q)}+\alpha _{5}\frac{G(x,x,x)G(y,q,q)}{1+G(x,p,p)+G(y,q,q)}\nonumber \\& \quad +\alpha _{6}\frac{G(p,p,p)G(x,p,p)}{1+G(x,p,p)+G(y,q,q)}+\alpha _{7}\frac{G(p,p,p)G(y,q,q)}{1+G(x,p,p)+G(y,q,q)}\nonumber \\& \quad +\alpha _{8}\frac{G(p,p,p)G(x,p,p)}{1+G(x,p,p)+G(y,q,q)}+\alpha _{9}\frac{G(p,p,p)G(y,q,q)}{1+G(x,p,p)+G(y,q,q)}\nonumber \\ & \Rightarrow G(x,p,p) \le \frac{\alpha _{1}}{2}[G(x,p,p)+G(y,q,q)]+\alpha _{2}G(x,p,p)+\alpha _{3}G(x,p,p)\nonumber \\& \Rightarrow (1-\frac{\alpha _{1}}{2}-\alpha _{2}-\alpha _{3})G(x,p,p) \le \frac{\alpha _{1}}{2}G(y,q,q)\nonumber \\ & \Rightarrow G(x,p,p) \le \frac{\alpha _{1}}{2-\alpha _{1}-2\alpha _{2}-2\alpha _{3}}G(y,q,q) \end{aligned}$$

Similarly,

5 $$\begin{aligned} G(y,q,q) \le \frac{\alpha _{1}}{2-\alpha _{1}-2\alpha _{2}-2\alpha _{3}}G(x,p,p) \end{aligned}$$

Adding (4) and (5) we have

$$\begin{aligned}&G(x,p,p)+G(y,q,q) \le \frac{\alpha _{1}}{2-\alpha _{1}-2\alpha _{2}-2\alpha _{3}}[G(x,p,p)+G(y,q,q)]\\&\quad \Rightarrow \left[ 1-\frac{\alpha _{1}}{2-\alpha _{1}-2\alpha _{2}-2\alpha _{3}}\right] [G(x,p,p)+G(y,q,q)] \le 0 \\&\quad \Rightarrow \frac{2(1-\alpha _{1}-\alpha _{2}-\alpha _{3})}{2-\alpha _{1}-2\alpha _{2}-2\alpha _{3}}[G(x,p,p)+G(y,q,q)] \le 0 \end{aligned}$$

Since $$\alpha _{1}+\alpha _{2}+\alpha _{3}<1$$, $$\frac{2(1-\alpha _{1}-\alpha _{2}-\alpha _{3})}{2-\alpha _{1}-2\alpha _{2}-2\alpha _{3}}>0$$.

Hence $G(x,p,p)+G(y,q,q)=0$, which implies that $x=p$ and $y=q \Rightarrow (x,y)=(p,q)$.

Thus *S*, *T*, *R* have unique coupled common fixed point.

This completes the proof. $\square$

### **Corollary 17**

*Let* (*X*, *G*) *be a complete symmetric*$G_{b}$-*metric space with parameter*$s\ge 1$*and let the mapping*$S:X^{2}\rightarrow X$*satisfying*

$$\begin{aligned} G(S(x,y),S(u,v),S(a,b))& \le \alpha _{1}\frac{G(x,u,a)+G(y,v,b)}{2} \\& \quad + \alpha _{2}\frac{G(S(x,y),S(u,v),S(a,b))G(x,u,a)}{1+G(x,u,a)+G(y,v,b)}\\& \quad + \alpha _{3}\frac{G(S(x,y),S(u,v)),S(a,b))G(y,v,b)}{1+G(x,u,a)+G(y,v,b)}\\& \quad +\alpha _{4}\frac{G(x,x,S(x,y))G(x,u,a)}{1+G(x,u,a)+G(y,v,b)}+\alpha _{5}\frac{G(x,x,S(x,y))G(y,v,b)}{1+G(x,u,a)+G(y,v,b)}\\& \quad +\alpha _{6}\frac{G(u,u,S(u,v))G(x,u,a)}{1+G(x,u,a)+G(y,v,b)}+\alpha _{7}\frac{G(u,u,S(u,v))G(y,v,b)}{1+G(x,u,a)+G(y,v,b)}\\& \quad +\alpha _{8}\frac{G(a,a,S(a,b))G(x,u,a)}{1+G(x,u,a)+G(y,v,b)}+\alpha _{9}\frac{G(a,a,S(a,b))G(y,v,b)}{1+G(x,u,a)+G(y,v,b)} \end{aligned}$$

*for all*$x,y,u,v,a,b \in X$*and*$\alpha _{1},\alpha _{2},\dots ,\alpha _{9}\ge 0$*with*$\alpha _{1}+\alpha _{2}+\alpha _{3}+2(\alpha _{4}+\alpha _{5})+\alpha _{6}+\alpha _{7}+\alpha _{8}+\alpha _{9}<1$. *ThenShas a unique coupled fixed point inX*.

### **Theorem 18**

*Let* (*X*, *G*) *be a complete symmetric*$G_{b}$-*metric space with parameter*$s\ge 1$*and let the mappings*$S,T,R:X\times X\rightarrow X$*satisfy*

6 $$\begin{aligned}&G(S(x,y),T(u,v),R(a,b)) \le \beta _{1}\frac{G(x,u,a)+G(y,v,b)}{2} \nonumber \\&\qquad +\beta _{2}\frac{G(x,x,S(x,y))G(u,u,T(u,v))}{1+s[G(x,x,T(u,v))+G(u,u,S(x,y))+G(x,u,a)+G(y,v,b)]} \nonumber \\&\qquad +\beta _{3}\frac{G(u,u,T(u,v)),G(a,a,R(a,b))}{1+s[G(u,u,R(a,b))+G(a,a,T(u,v))+G(x,u,a)+G(y,v,b)]} \nonumber \\&\qquad +\beta _{4}\frac{G(a,a,R(a,b))G(x,x,S(x,y))}{1+s[G(a,b,S(x,y))+G(x,x,R(a,b))+G(x,u,a)+G(y,v,b)]} \end{aligned}$$

*for all*$x,y,u,v,a,b \in X$*and*$\beta _{1},\beta _{2},\beta _{3},\beta _{4}$*are non-negative real numbers with*$\beta _{1}+\beta _{2}+\beta _{3}+\beta _{4}<1$. *ThenS*, *T*, *Rhave unique coupled common fixed point*.

### *Proof*

Let $x_{0},y_{0}\in X$ be arbitrary points. Define

$$\begin{aligned} x_{3k+1}& = S(x_{3k},y_{3k}), y_{3k+1}=S(y_{3k},x_{3k})\\ x_{3k+2}& = T(x_{3k+1},y_{3k+1}), y_{3k+2}=T(y_{3k+1},x_{3k+1})\\ x_{3k+3}& = R(x_{3k+2},y_{3k+2}), y_{3k+3}=R(y_{3k+2},x_{3k+2}) \end{aligned}$$

for $$k=0,1,2,3 \ldots$$

Then

$$\begin{aligned}&G(x_{3k+1},x_{3k+2},x_{3k+3})= G(S(x_{3k},y_{3k}),T(x_{3k+1},y_{3k+1}),R(x_{3k+1},y_{3k+1})) \\&\quad \le \beta _{1}\frac{G(x_{3k},x_{3k+1},x_{3k+2})+G(y_{3k},y_{3k+1},y_{3k+2})}{2} \\&\qquad +\beta _{2}\frac{G(x_{3k},x_{3k},S(x_{3k},y_{3k}))G(x_{3k+1},x_{3k+1},T(x_{3k+1},y_{3k+1}))}{1+s[G(x_{3k},x_{3k},T(x_{3k+1},y_{3k+1}))+G(x_{3k+1},x_{3k+1},S(x_{3k},y_{3k}))+G(x_{3k},x_{3k+1},x_{3k+2})+G(y_{3k},y_{3k+1},y_{3k+2})]} \\&\qquad +\beta _{3}\frac{G(x_{3k+1},x_{3k+1},T(x_{3k+1},y_{3k+1}))G(x_{3k+2},x_{3k+2},R(x_{3k+2},y_{3k+2}))}{1+s[G(x_{3k+1},x_{3k+1},R(x_{3k+2},y_{3k+2}))+G(x_{3k+2},x_{3k+2},T(x_{3k+1},y_{3k+1}))+G(x_{3k},x_{3k+1},x_{3k+2})+G(y_{3k},y_{3k+1},y_{3k+2})]} \\&\qquad +\beta _{4}\frac{G(x_{3k+2},x_{3k+2},R(x_{3k+2},y_{3k+2}))G(x_{3k},x_{3k},S(x_{3k},y_{3k}))}{1+s[G(x_{3k+2},x_{3k+2},S(x_{3k},y_{3k}))+G(x_{3k},x_{3k},R(x_{3k+2},y_{3k+2}))+G(x_{3k},x_{3k+1},x_{3k+2})+G(y_{3k},y_{3k+1},y_{3k+2})]}\\&\quad =\beta _{1}\frac{G(x_{3k},x_{3k+1},x_{3k+2})+G(y_{3k},y_{3k+1},y_{3k+2})}{2}\\&\qquad +\beta _{2}\frac{G(x_{3k},x_{3k},x_{3k+1})G(x_{3k+1},x_{3k+1},x_{3k+2})}{1+s[G(x_{3k},x_{3k},x_{3k+2})+G(x_{3k+1},x_{3k+1},x_{3k+1})+G(x_{3k},x_{3k+1},x_{3k+2})+G(y_{3k},y_{3k+1},y_{3k+2})]} \\&\qquad +\beta _{3}\frac{G(x_{3k+1},x_{3k+1},x_{3k+2})G(x_{3k+2},x_{3k+2},x_{3k+3})}{1+s[G(x_{3k+1},x_{3k+1},x_{3k+3})+G(x_{3k+2},x_{3k+2},x_{3k+2})+G(x_{3k},x_{3k+1},x_{3k+2})+G(y_{3k},y_{3k+1},y_{3k+2})]} \\&\qquad +\beta _{4}\frac{G(x_{3k+2},x_{3k+2},x_{3k+3})G(x_{3k},x_{3k},x_{3k+1})}{1+s[G(x_{3k+2},x_{3k+2},x_{3k+1})+G(x_{3k},x_{3k},x_{3k+3})+G(x_{3k},x_{3k+1},x_{3k+2})+G(y_{3k},y_{3k+1},y_{3k+2})]} \\&\quad \le \beta _{1}\frac{G(x_{3k},x_{3k+1},x_{3k+2})+G(y_{3k},y_{3k+1},y_{3k+2})}{2}\\&\qquad +\beta _{2}\frac{G(x_{3k},x_{3k+1},x_{3k+2})G(x_{3k+1},x_{3k+2},x_{3k+3})}{1+s[G(x_{3k},x_{3k},x_{3k+2})+G(x_{3k},x_{3k+1},x_{3k+2})+G(y_{3k},y_{3k+1},y_{3k+2})]} \\&\qquad +\beta _{3}\frac{G(x_{3k},x_{3k+1},x_{3k+2})G(x_{3k+1},x_{3k+2},x_{3k+3})}{1+s[G(x_{3k+1},x_{3k+1},x_{3k+3})+G(x_{3k},x_{3k+1},x_{3k+2})+G(y_{3k},y_{3k+1},y_{3k+2})]} \\&\qquad +\beta _{4}\frac{G(x_{3k+1},x_{3k+2},x_{3k+3})G(x_{3k},x_{3k+1},x_{3k+2})}{1+s[G(x_{3k+2},x_{3k+2},x_{3k+1})+G(x_{3k},x_{3k},x_{3k+3})+G(x_{3k},x_{3k+1},x_{3k+2})+G(y_{3k},y_{3k+1},y_{3k+2})]} \\&\quad \le \beta _{1}\frac{G(x_{3k},x_{3k+1},x_{3k+2})+G(y_{3k},y_{3k+1},y_{3k+2})}{2}\\&\qquad +\beta _{2} G(x_{3k},x_{3k+1},x_{3k+2})+\beta _{3} G(x_{3k},x_{3k+1},x_{3k+2})+\beta _{4} G(x_{3k},x_{3k+1},x_{3k+2}) \\&\quad \le \beta _{1}\frac{G(x_{3k},x_{3k+1},x_{3k+2})+G(y_{3k},y_{3k+1},y_{3k+2})}{2} \\&\qquad +(\beta _{2}+\beta _{3}+\beta _{4})G(x_{3k+1},x_{3k+2},x_{3k+3}) \end{aligned}$$

which implies that

7 $$\begin{aligned} (1-\beta _{2}-\beta _{3}-\beta _{4})G(x_{3k+1},x_{3k+2},x_{3k+3})& \le \frac{\beta _{1}}{2} G(x_{3k},x_{3k+1},x_{3k+2}) +\frac{\beta _{1}}{2} G(y_{3k},y_{3k+1},y_{3k+2}) \nonumber \\ \Rightarrow G(x_{3k+1},x_{3k+2},x_{3k+3})& \le \frac{\beta _{1}}{2(1-\beta _{2}-\beta _{3}-\beta _{4})} [G(x_{3k},x_{3k+1},x_{3k+2}) \nonumber \\&\quad +G(y_{3k},y_{3k+1},y_{3k+2})] \end{aligned}$$

Similarly we can show that

8 $$\begin{aligned} (1-\beta _{2}-\beta _{3}-\beta _{4})G(y_{3k+1},y_{3k+2},y_{3k+3})& \le \frac{\beta _{1}}{2} G(x_{3k},x_{3k+1},x_{3k+2}) +\frac{\beta _{1}}{2} G(y_{3k},y_{3k+1},y_{3k+2}) \nonumber \\ \Rightarrow G(y_{3k+1},y_{3k+2},y_{3k+3})& \le \frac{\beta _{1}}{2(1-\beta _{2}-\beta _{3}-\beta _{4})} [G(x_{3k},x_{3k+1},x_{3k+2}) \nonumber \\&\quad +G(y_{3k},y_{3k+1},y_{3k+2})] \end{aligned}$$

Adding (7) and (8) we have

$$\begin{aligned} G(x_{3k+1},x_{3k+2},x_{3k+3}) + & G(y_{3k+1},y_{3k+2},y_{3k+3}) \\& \le\; \frac{\beta _{1}}{1-\beta _{2}-\beta _{3}-\beta _{4}}[G(x_{3k},x_{3k+1},x_{3k+2})+G(y_{3k},y_{3k+1},y_{3k+2})]\\& =\; k [G(x_{3k},x_{3k+1},x_{3k+2})+G(y_{3k},y_{3k+1},y_{3k+2})] \end{aligned}$$

where $$k=\frac{\beta _{1}}{1-\beta _{2}-\beta _{3}-\beta _{4}}$$.

Also, we can show that

$$\begin{aligned} & G(x_{3k+2},x_{3k+3},x_{3k+4}) + G(y_{3k+2},y_{3k+3},y_{3k+4}) \\& \quad \le k [G(x_{3k+1},x_{3k+2},x_{3k+3})+G(y_{3k+1},y_{3k+2},y_{3k+3})] \\& \quad \le k^{2}[G(x_{3k},x_{3k+1},x_{3k+2})+G(y_{3k},y_{3k+1},y_{3k+2})] \end{aligned}$$

Now if $G(x_{n},x_{n+1},x_{n+2})+G(y_{n},y_{n+1},y_{n+2})=G_{n}$ then $G_{n}\le kG_{n-1}\le \cdots \le k^{n}G_{0}$.

By property (3) of Definition 1, we have

$$\begin{aligned} G(x_{n},x_{n+1},x_{n+1})+G(y_{n},y_{n+1},y_{n+1}) \le G_{n} \le k^{n}G_{0} \end{aligned}$$

So, for $m>n$ we have

$$\begin{aligned} G(x_{n},x_{m},x_{m}) & + G(y_{n},y_{m},y_{m}) \\& \le s[G(x_{n},x_{n+1},x_{n+1})+G(x_{n+1},x_{m},x_{m})+G(y_{n},y_{n+1},y_{n+1})+G(y_{n+1},y_{m},y_{m})]\\& \le s[G(x_{n},x_{n+1},x_{n+1})+G(y_{n},y_{n+1},y_{n+1})] \\& \quad +s^{2}[G(x_{n+1},x_{n+2},x_{n+2})+G(y_{n+1},y_{n+2},y_{n+2})] \\& \quad + \cdots +s^{m-n}[G(x_{m-1},x_{m},x_{m})+G(y_{m-1},y_{m},y_{m})] \\& \le sk^{n}G_{0}+s^{2}k^{n+1}G_{0}+ \cdots +s^{m-n}k^{m-1}G_{0} \\& < sk^{n}[1+(sk)+(sk)^{2}+ \cdots ]G_{0} \\& = \frac{sk^{n}}{1-sk}G_{0} \rightarrow 0 \quad {\rm as}\;n \rightarrow \infty \end{aligned}$$

Therefore $\{x_{n}\}$ and $\{y_{n}\}$ are Cauchy sequences in *X*. Since *X* is complete $G_{b}$-metric space, there exist $x,y \in X$ such that $x_{n} \rightarrow x$ and $y_{n} \rightarrow y$ as $n \rightarrow \infty$.

Now, we will show that $x=S(x,y)$ and $y=S(y,x)$. Suppose on contrary that $x\not =S(x,y)$ and $y\not =S(y,x)$, so that $G(x,S(x,y),S(x,y))=l_{1}>0$ and $G(y,y,S(y,x))=l_{2}>0$. Consider the following and using inequality (6), we get

$$\begin{aligned} l_{1}& = G(x,S(x,y),S(x,y)) \\& \le s[G(x,x_{n+1},x_{n+1})+G(x_{n+1},S(x,y),S(x,y))] \\& = s[G(x,x_{n+1},x_{n+1})+G(S(x_{n},y_{n}),S(x,y),S(x,y))] \\& \le sG(x,x_{n+1},x_{n+1})+s\left[ \beta _{1}\frac{G(x_{n},x,x)+G(y_{n},y,y)}{2} \right. \\& \quad \left. +\beta _{2}\frac{G(x_{n},x_{n},S(x_{n},y_{n}))G(x,x,S(x,y))}{1+s[G(x_{n},x_{n},S(x,y))+G(x,x,S(x_{n},y_{n}))+G(x_{n},x,x)+G(y_{n},y,y)]} \right. \\& \quad \left. +\beta _{3}\frac{G(x,x,S(x,y))G(x,x,S(x,y))}{1+s[G(x,x,S(x,y))+G(x,x,S(x,y))+G(x_{n},x,x)+G(y_{n},y,y)]} \right. \\& \quad \left. +\beta _{4}\frac{G(x,x,S(x,y))G(x_{n},x_{n},S(x_{n},y_{n}))}{1+s[G(x,x,S(x_{n},y_{n}))+G(x_{n},x_{n},S(x,y))+G(x_{n},x,x)+G(y_{n},y,y)]}\right] \\& = sG(x,x_{n+1},x_{n+1})+\frac{s\beta _{1}}{2}G(x_{n},x,x)+\frac{s\beta _{1}}{2}G(y_{n},y,y) \\& \quad +s\beta _{2}\frac{G(x_{n},x_{n},x_{n+1})G(x,x,S(x,y))}{1+s[G(x_{n},x_{n},S(x,y))+G(x,x,x_{n+1})+G(x_{n},x,x)+G(y_{n},y,y)]} \\& \quad +s\beta _{3}\frac{G(x,x,S(x,y))G(x,x,S(x,y))}{1+s[G(x,x,S(x,y))+G(x,x,S(x,y))+G(x_{n},x,x)+G(y_{n},y,y)]} \\& \quad +s\beta _{4}\frac{G(x,x,S(x,y))G(x_{n},x_{n},x_{n+1})}{1+s[G(x,x,x_{n+1})+G(x_{n},x_{n},S(x,y))+G(x_{n},x,x)+G(y_{n},y,y)]} \end{aligned}$$

Taking limit as $n \rightarrow \infty$ we get

$$\begin{aligned}&G(x,S(x,y),S(x,y)) \le 0+0+0+s.\beta _{2}.0+\beta _{3}G(x,x,S(x,y))+s.\beta _{4}.0 \\&\quad \Rightarrow G(x,S(x,y),S(x,y)) \le 2s\beta _{3}G(x,S(x,y),S(x,y)) \\&\quad \Rightarrow (1-2s\beta _{3})G(x,S(x,y),S(x,y)) \le 0 \end{aligned}$$

Therefore $$G(x,S(x,y),S(x,y))=0$$.

Which implies that $x=S(x,y)$. Similarly we can prove that $y=S(y,x)$.

Also, we can prove that $x=T(x,y)$, $y=T(y,x)$ and $x=R(x,y)$, $y=R(y,x)$.

Hence, (*x*, *y*) is a common coupled fixed point of *S*, *T* and *R*.

In order to prove the uniqueness of the common coupled fixed point of *S*, *T* and *R*, if possible let (*p*, *q*) be the second common copuled fixed point of *S*, *T* and *R*.

Then by using inequality (6) we have

9 $$\begin{aligned}&G(x,p,p) = G(S(x,y),T(p,q),R(p,q)) \nonumber \\&\quad \le \beta _{1}\frac{G(x,p,p)+G(y,q,q)}{2} \nonumber \\&\qquad +\beta _{2}\frac{G(x,x,S(x,y))G(p,p,T(p,q))}{1+s[G(x,x,T(p,q))+G(p,p,S(x,y))+G(x,p,p)+G(y,q,q)]} \nonumber \\&\qquad +\beta _{3}\frac{G(p,p,T(p,q))G(p,p,R(p,q))}{1+s[G(p,p,R(p,q))+G(p,p,T(p,q))+G(x,p,p)+G(y,q,q)]} \nonumber \\&\qquad +\beta _{4}\frac{G(p,p,R(p,q))G(x,x,S(x,y))}{1+s[G(p,p,S(x,y))+G(x,x,R(p,q))+G(x,p,p)+G(y,q,q)]} \nonumber \\&\quad \Rightarrow G(x,p,p) \le \beta _{1}\frac{G(x,p,p)+G(y,q,q)}{2}+\beta _{2}.0+\beta _{3}.0+\beta _{4}.0 \nonumber \\&\quad \Rightarrow \left( 1-\frac{\beta _{1}}{2}\right) G(x,p,p) \le \frac{\beta _{1}}{2}G(y,q,q) \nonumber \\&\quad \Rightarrow G(x,p,p) \le \frac{\beta _{1}}{2-\beta _{1}}G(y,q,q) \end{aligned}$$

Similarly

10 $$\begin{aligned} G(y,q,q) \le \frac{\beta _{1}}{2-\beta _{1}}G(x,p,p) \end{aligned}$$

Adding (9) and (10) we have

$$\begin{aligned}&G(x,p,p)+G(y,q,q) \le \frac{\beta _{1}}{2-\beta _{1}}[G(x,p,p)+G(y,q,q)] \\&\quad \Rightarrow \left( 1-\frac{\beta _{1}}{2-\beta _{1}}\right) [G(x,p,p)+G(y,q,q)] \le 0 \\&\quad \Rightarrow \frac{2-2\beta _{1}}{2-\beta _{1}}[G(x,p,p)+G(y,q,q)] \le 0 \end{aligned}$$

But $$\frac{2-2\beta _{1}}{2-\beta _{1}} > 0$$. Therefore $$G(x,p,p)+G(y,q,q)=0$$

Which implies that $$x=p$$ and $$y=q$$$$\Rightarrow (x,y)=(p,q)$$.

Thus *S*, *T*, *R* have a unique common coupled fixed point.

This completes the proof. $\square$

### **Corollary 19**

*Let* (*X*, *G*) *be a complete symmetric*$G_{b}$-*metric space with parameter*$s\ge 1$*and let the mapping*$S:X^{2} \rightarrow X$*satisfying*

$$\begin{aligned} G(S(x,y),S(u,v),S(a,b))& \le \beta _{1}\frac{G(x,u,a)+G(y,v,b)}{2} \\& \quad +\beta _{2}\frac{G(x,x,S(x,y))G(u,u,S(u,v))}{1+s[G(x,x,S(u,v))+G(u,u,S(x,y))+G(x,u,a)+G(y,v,b)]} \\& \quad +\beta _{3}\frac{G(u,u,S(u,v))G(a,a,S(a,b))}{1+s[G(u,u,S(a,b))+G(a,a,S(u,v))+G(x,u,a)+G(y,v,b)]} \\& \quad +\beta _{4}\frac{G(a,a,S(a,b))G((x,x,S(x,y))}{1+s[G(a,a,S(x,y))+G(x,x,S(a,b))+G(x,u,a)+G(y,v,b)]} \\ \end{aligned}$$

*for all*$x,y,u,v,a,b \in X$*and*$\beta _{1},\beta _{2},\beta _{3},\beta _{4}$*are non-negative realnumbers with*$\beta _{1}+\beta _{2}+\beta _{3}+\beta _{4}< 1$. *ThenShas a unique coupled fixed point*.

### *Example 20*

Let $X=[0,1]$, $G:X^{3} \rightarrow R$ be defined by

$$\begin{aligned} G(x,y,z)=\max \{d(x,y),d(y,z),d(z,x)\}\;{\text{with }} d(x,y)=|x-y|^{2} {\text { where }} s=2. \end{aligned}$$

Define

$$\begin{aligned} S(x,y)& = \frac{x-y}{6}\\ T(u,v)& = \frac{u-v}{12}\\ R(a,b)& = \frac{a-b}{24} \end{aligned}$$

for all $x,y,u,v,a,b \in X$. Suppose $\alpha =\frac{1}{4}$ and $0< \alpha _{2},\alpha _{3},\alpha _{4},\alpha _{5},\alpha _{6},\alpha _{7},\alpha _{8},\alpha _{9}<1$. Now we have

$$\begin{aligned} G(S(x,y),T(u,v),R(a,b))& = G\left( \frac{x-y}{6},\frac{u-v}{12},\frac{a-b}{24}\right) \\& = \max \left\{ \left| \frac{2(x-y)-(u-v)}{12}\right| ^{2},\left| \frac{2(u-v)-(a-b)}{24}\right| ^{2}, \quad \left| \frac{4(x-y)-(a-b)}{24}\right| ^{2}\right\} \end{aligned}$$

Let $$\max =\frac{2(x-y)-(u-v)}{12}$$, then

$$\begin{aligned} G\left( \frac{x-y}{6},\frac{u-v}{12},\frac{a-b}{24}\right)& = \left| \frac{2(x-y)-(u-v)}{12}\right| ^{2} \\& \le \frac{1}{4}\left\{ \frac{G(x,u,a)+G(y,v,b)}{2}\right\} \\& \le \frac{1}{4}\left\{ \frac{G(x,u,a)+G(y,v,b)}{2}\right\} +\alpha _{2}\frac{G(S(x,y),T(u,v),R(a,b))G(x,u,a)}{1+G(x,u,a)+G(y,v,b)}\\& \quad +\alpha _{3}\frac{G(S(x,y),T(u,v),R(a,b))G(y,v,b)}{1+G(x,u,a)+G(y,v,b)} +\alpha _{4}\frac{G(x,x,S(x,y))G(x,u,a)}{1+G(x,u,a)+G(y,v,b)} \\& \quad +\alpha _{5}\frac{G(x,x,S(x,y))G(y,v,b)}{1+G(x,u,a)+G(y,v,b)} +\alpha _{6}\frac{G(u,u,T(u,v))G(x,u,a)}{1+G(x,u,a)+G(y,v,b)} \\& \quad +\alpha _{7}\frac{G(u,u,T(u,v))G(y,v,b)}{1+G(x,u,a)+G(y,v,b)} +\alpha _{8}\frac{G(a,a,R(a,b))G(x,u,a)}{1+G(x,u,a)+G(y,v,b)} \\& \quad +\alpha _{9}\frac{G(a,a,R(a,b))G(y,v,b)}{1+G(x,u,a)+G(y,v,b)} \end{aligned}$$

Obviously all the conditions of Theorem 16 are satisfied. Also, (0, 0) is the unique common coupled fixed point of *S*, *T* and *R*.

## Conclusion

We prove the existence and uniqueness of common coupled fixed point theorems for three mappings with a new rational contractive conditions in $G_{b}$-metric space. Our results improve and genaralise the similar results in *b*-metric and *G*-metric spaces. These results may be extended to other spaces.
